# Physical enhancement of older adults using hyperbaric oxygen: a randomized controlled trial

**DOI:** 10.1186/s12877-024-05146-3

**Published:** 2024-07-03

**Authors:** Amir Hadanny, Efrat Sasson, Laurian Copel, Malka Daniel-Kotovsky, Eldad Yaakobi, Erez Lang, Gregory Fishlev, Nir Polak, Mony Friedman, Keren Doenyas, Shachar Finci, Yonatan Zemel, Yair Bechor, Shai Efrati

**Affiliations:** 1 Sagol Center for Hyperbaric Medicine and Research, Shamir (Assaf Harofeh) Medical Center, Zerifin, 70300 Israel; 2 School of Medicine, Faculty of Medical and Health Sciences, Tel-Aviv University, Tel-Aviv, Israel; 3 Radiology Department, Shamir Medical Center, Zerifin, Israel; 4 Research and Development Unit, Shamir Medical Center, Zerifin, Israel; 5 Sagol School of Neuroscience, Tel-Aviv University, Tel-Aviv, Israel

**Keywords:** Hyperbaric oxygen therapy, Aging, Oxygen consumption, Cardiac perfusion, Physical performance, Endurance, Cardiac MRI

## Abstract

**Introduction:**

Aging is associated with a progressive decline in the capacity for physical activity. The objective of the current study was to evaluate the effect of an intermittent hyperbaric oxygen therapy (HBOT) protocol on maximal physical performance and cardiac perfusion in sedentary older adults.

**Methods:**

A randomized controlled clinical trial randomized 63 adults (> 64yrs) either to HBOT (*n* = 30) or control arms (*n* = 33) for three months. Primary endpoint included the maximal oxygen consumption (VO_2_Max) and VO_2_Max/Kg, on an E100 cycle ergometer. Secondary endpoints included cardiac perfusion, evaluated by magnetic resonance imaging and pulmonary function. The HBOT protocol comprised of 60 sessions administered on a daily basis, for 12 consecutive weeks, breathing 100% oxygen at 2 absolute atmospheres (ATA) for 90 min with 5-minute air breaks every 20 min.

**Results:**

Following HBOT, improvements were observed in VO_2_Max/kg, with a significant increase of 1.91 ± 3.29 ml/kg/min indicated by a net effect size of 0.455 (*p* = 0.0034). Additionally, oxygen consumption measured at the first ventilatory threshold (VO_2_VT_1_) showed a significant increase by 160.03 ± 155.35 ml/min (*p* < 0.001) with a net effect size of 0.617. Furthermore, both cardiac blood flow (MBF) and cardiac blood volume (MBV) exhibited significant increases when compared to the control group. The net effect size for MBF was large at 0.797 (*p* = 0.008), while the net effect size for MBV was even larger at 0.896 (*p* = 0.009).

**Conclusion:**

The findings of the study indicate that HBOT has the potential to improve physical performance in aging adults. The enhancements observed encompass improvements in key factors including VO_2_Max, and VO_2_VT_1_. An important mechanism contributing to these improvements is the heightened cardiac perfusion induced by HBOT.

**Trial registration:**

ClinicalTrials.gov Identifier NCT02790541 (registration date 06/06/2016).

## Introduction

Aging is associated with a progressive decline in physical activity capacity, even in healthy individuals who exercise regularly and have no major illnesses or musculoskeletal issues. Moreover, the rate of performance decline is accelerated from the age of 70 years onwards [1, 2]. Central to this decline is a decrease in the maximal oxygen consumption (VO_2_Max), with more than 1% loss per year. The decrease in VO_2_Max is a major contributor to frailty and age-related loss of function, affecting 25–50% of individuals over 85 [3, 4]. Apart from being a main limiting factor of endurance performance, VO_2_Max is a strong predictor of mortality and cardiovascular morbidity [5, 6].

The age related decline in VO_2_Max is attributed to a decline in cardiovascular efficiency due to reduced maximal cardiac output and its maldistribution, decreased pulmonary function, and a decrease in muscle mass and muscle oxidative capacity decrease due to mitochondrial dysfunction [7–10]. Engaging in high levels of physical exercise throughout one’s life can help to slow down the multi-systemic deterioration that is commonly observed in inactive individuals. However, even in athletes the accelerated decline in performance occurs as well after the age of 70 [2].

Hyperbaric oxygen therapy (HBOT) involves administering 100% oxygen at an environmental pressure higher than one absolute atmosphere (ATA). It is now realized that the combined action of both hyperoxia and hyperbaric pressure, leads to significant improvement in tissue oxygenation while targeting both oxygen and pressure sensitive genes, resulting in improved mitochondrial metabolism with anti-apoptotic and anti-inflammatory effects [11–13]. Moreover, these genes induce stem cell proliferation and augment circulating levels of endothelial progenitor cells (EPCs) and angiogenesis factors, which induce angiogenesis and improved blood flow in the ischemic area [14, 15].

Certain HBOT protocols, that include repeated intermittent exposure to hyperoxia at high atmospheric pressure, can induce physiological effects similar to those seen during hypoxia in a hyperoxic environment [11, 12, 14, 15]. In our previous clinical trial on middle-aged master athletes, HBOT enhanced physical performance including VO_2_Max [16]. The main mechanisms demonstrated were an increase in both mitochondrial function and mitochondria count per muscle fiber (biogenesis) [16]. On another study, we reported significant cognitive function enhancement with improved cerebral blood flow [17]. HBOT induced angiogenesis and enhanced tissue perfusion in healthy aging populations is not limited to the brain as was demonstrated directly by skin biopsies [18]. With regards to cardiac function, HBOT was found to improve echocardiographic parameters in aging asymptomatic individuals [19]. However, the effects of HBOT on physical performance and cardiac perfusion in non-athletic elderly population has yet to be investigated.

The aim of the current study was to evaluate the effect of an intermittent HBOT protocol on maximal physical performance and cardiac perfusion in sedentary older adults.

## Methods

### Study design

A prospective, randomized controlled study of aging adults. The study was conducted between 2016 and 2020 and the protocol was approved by the Shamir medical center institutional review board. The study was registered in the national institutes of health (NIH) clinical trials registry, number NCT02790541 (registration date 06/06/2016). The study was performed in Shamir medical center. All methods were performed in accordance with the relevant guidelines and regulations of the Declaration of Helsinki.

### Subjects

Seventy non-athletes adults without pathological cognitive decline, aged 64 and older, who lived independently in good functional and cognitive status were enrolled. Exclusion criteria included: previous treatment with HBOT for any reason during the last three months, any history of malignancy during the last year, any pathological cognitive decline, severe chronic renal failure (glomerular filtration rate < 30), uncontrolled diabetes mellitus (HbA1C > 8, fasting glucose > 200), immunosuppressants, magnetic resonance imaging (MRI) contraindications, active smoking and pulmonary diseases.

All subjects were requested to continue their current lifestyle, diet and physical training regimen (if any), with no changes in volume or training intensity during the study.

### Randomization and masking

After signing an informed consent, the subjects were randomly assigned (1:1) to either the HBOT or the control (no intervention) groups. Randomization sequence was performed manually by the study coordinator (MDK) and concealed allocation was ensured. Patients were not blinded to the allocated group. Assessors were blinded to the subjects’ intervention assignment. All data was stored in a dedicated database and were checked for accuracy and completeness.

### Interventions

The HBOT protocol was administrated in a multiplace Starmed-2700 chamber (HAUX, Germany). The protocol comprised of 60 sessions, administered on a daily basis, five sessions per week, for 12 consecutive weeks, within a three-month period. Each session included breathing 100% oxygen by mask at 2ATA for 90 min with 5-minute air breaks every 20 min. Compression/ decompression rates were 1 meter per minute.

The control arm received no active intervention. During the trial, neither lifestyle, physical training and diet changes, nor medications adjustments were allowed for either group.

### Outcomes

The subjects were evaluated at baseline (up to1-2 weeks prior to their interventional protocol) and 1–2 weeks after the last HBOT session or control period.

#### Cardiopulmonary maximal exercise test (CPET)

Exercise tests were measured using an E100 cycle ergometer (COSMED, Rome, Italy). Subjects who did not complete the test on this machine were excluded from analysis. Gas exchange was measured by a Quark CPET system (COSMED), with breath-by-breath sampling technology and integrated heartrate and exercise electrocardiogram (ECG) monitoring and recording with a 12-lead ECG system (COSMED, Rome, Italy). Data were collected on a dedicated computer using the Omnia Metabolic Modules software (COSMED). Before each test, the gas analyzers and flow meter were calibrated.

The start of the protocol included a one-minute rest without pedaling, followed by a two-minute warm up. The testing protocol included a ramp power increase of 15 watts every minute (1 W every 4 s) starting from 0 watts, while pedaling cadence had to be maintained at 70 revolutions per minute (RPM). Exhaustion was reached when cadence could not be maintained above 70 RPM or when a participant terminated the test. Following exhaustion, participants underwent a three-minute recovery period with no pedaling (0 W).

A blinded physiologist performed analysis of each CPET test separately, masked from the individual name, group allocation, date of performance and whether the test was a baseline or post-intervention measurement. The breath-by-breath dataset was averaged in epochs of seven breaths. VO_2_Max and VO_2_VT_1_ (oxygen uptake at the first ventilatory threshold) were determined by validated criteria. For VO_2_Max, a plateau or decline pattern in VO_2_, with increasing work rate while pedaling cadence maintained at 70 RPM, for at least 30 s. For VO_2_VT_1_, the combination of three validated methods with agreement: (1) the ventilatory equivalents method (ventilation (VE)/VO_2_ and VE/VCO_2_ to work rate) (2) End tidal O_2_ pressure method (PETO_2_ to work rate) (3) Modified V slope method (VCO_2_ to VO_2_) [20]. Compared parameters were maximal power output, VO_2_Max, VO_2_Max/Work Rate (WR), VO_2_Max /heartrate, VO_2_VT_1_, breathing reserve (BR), respiratory quotient (RQ), heartrate, VE and volume of CO_2_ expired (VCO_2_).

#### Pulmonary function

Measurements of pulmonary functions were performed using the KoKo Sx1000 spirometer (Nspire health, USA). The equipment was calibrated using a 3-l syringe before performing measurements according to the manufacturer’s instructions. Measurements were performed by a trained technician. The forced expiratory maneuvers were performed as recommended by the guidelines [21].

The forced vital capacity (FVC), forced expiratory volume in 1 s (FEV1), the Tiffeneau-Pinelli index (FEV1/FVC), and peak expiratory flow rate (PEF) were taken as the highest readings obtained from at least three satisfactory forced expiratory maneuvers. Mean forced mid-expiratory flow rate (FEF25–75%) and forced expiratory flow rates at 25, 50 and 75% of FVC expired (FEF25%, FEF50% and FEF75%) were taken as the best values from flow–volume loops not differing by > 5% from the highest FVC.

#### Cardiac MRI

MRI scans were performed on a MAGNETOM AERA 1.5T scanner (Siemens, Erlangen, Germany). MRI first-pass perfusion imaging was performed to quantify myocardial blood flow (MBF) and volume (MBV). T1-Trufisp sequence was performed before and after injection of 0.1mmol of Dotarem. 50 repetitions were performed. Scanning was gated to the cardiac cycle. Sequence parameters of each repetition were: repetition time (TR) 178.3 miliseconds (ms), echo time (TE) 0.95 ms, Inversion time (TI) 110 ms, Flip angle 50°, in-plain spatial resolution 160 × 125, slice thickness 8 mm with 8 mm spacing, pixel size 2.875 × 2.875, 7 slices. MRI Analysis included rigid motion correction using SPM software (version 12, UCL, London, UK). Signal intensity–time data were converted to concentration-time data by subtracting the baseline signal. Arterial input function (AIF) was measured by an ROI drawn in the left ventricle.

MBF and MBV were calculated using in-house software written in MATLAB 2018b (MathWorks, Natick, MA) using deconvolution. To adjust for variability between scans, MBF and MBV maps were normalized. The normalization was performed by dividing pixel by pixel MBF and MBV maps by an average of region of interest (ROI) drawn manually in the left ventricle allowing measuring peak intensity. ROI analysis was performed to extract the normalized values of MBF and MBV in the myocardium.

#### Fat Mass

Fat mass was evaluated using a standard dual X-ray absorptiometry (DXA) machine (GE, USA) DXA according to the manufacture standard protocol [22]. The values for fat percent (%) for whole body were measured. Scan mode selection was automatically processed by the manufacturer’s software for GE.

#### Safety

Subjects were monitored for adverse events including barotraumas (either ear or sinuses) and oxygen toxicity (pulmonary and central nervous system).

### Statistical analysis

Continuous data are expressed as means ± standard-deviation. Normal distributions for all variables were tested using the Kolmogorov-Smirnov test. Unpaired and paired t-tests were performed to compare baseline measurements variables between the two groups. Net effect sizes were evaluated using Cohen’s d method. Cohen’s classification of effect sizes delineates them as small (d = 0.2), medium (d = 0.5), and large (d ≥ 0.8) [23]. Categorical data are expressed in frequencies and percentages and were compared by chi-square tests. CPET missing data were imputed using the multiple imputation approach using five iterations pooled data.

Univariate analyses were performed using Chi-Square/Fisher’s exact test to identify significant confounders. Out of the baseline parameters, age was the only confounder (p-0.08). To evaluate HBOT’s effects on physical performance, normality, homoscedasticity, and linearity tests were considered as basic assumptions for the use of t tests and analysis of covariance (ANCOVA) to control for the baseline value of VO_2_Max and adjust for possible confounding variable age.

Pearson’s correlations were used between MBF and MBV and CPET parameters. Correlation coefficients were interpreted as follows: negligible (0-0.1), weak (0.1–0.39), moderate (0.4–0.69), strong (0.7–0.89), and very strong (0.9-1) [24].

The statistical significance threshold was set to 0.05. Data were statistically analyzed using MATLAB 2018b (MathWorks, Natick, MA).

#### Sample size

Sample size for this clinical study was calculated for a primary endpoint of cognitive function as been published previously [17] The post-hoc power calculation for the current study primary endpoint was performed. Using an alpha of 0.05, total sample size of 63 and effect size of 0.341, with a repeated measures within-between interaction ANOVA design, a power of 0.999 was found.

## Results

**Fig. 1:**
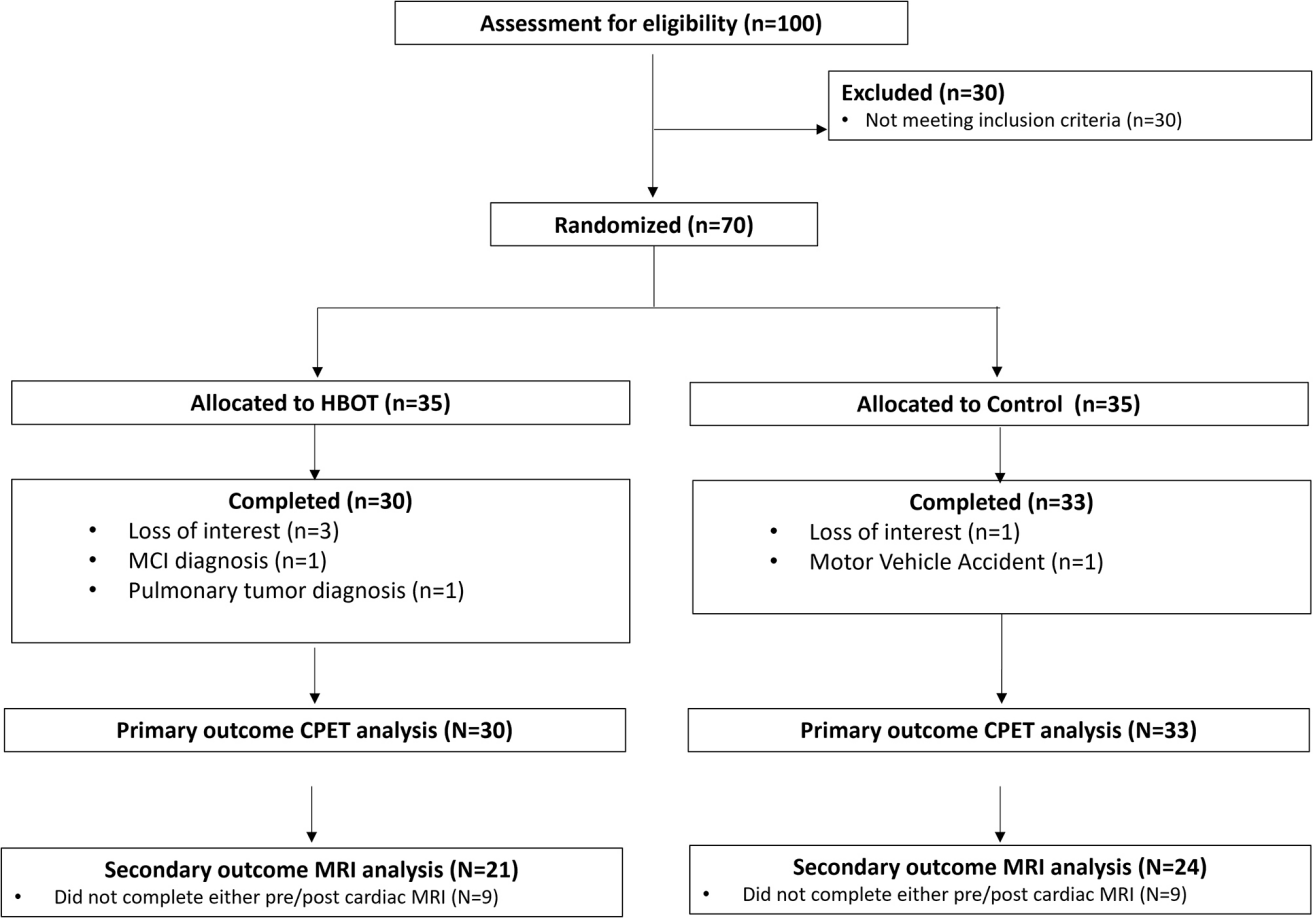
Participants’ flowchart

Out of 100 individuals that contacted for participation, 70 were eligible and signed an informed consent. Seven dropped out prior to completion of protocol (2 in the control and 5 in the HBOT arms). Thus, a total of 63 individuals completed HBOT or control interventions. Eight individuals (3 from HBOT and 4 from the control arms) did not complete the post intervention exercise test on the E100 cycle ergometer device due to technical issues with the machine, and their data were imputed. The CPET analysis was conducted on 30 HBOT and 33 control patients. (Figure 1). Out of the these, 18 did not complete either pre/post cardiac MRI scans (9 from HBOT and 9 from the control arms). A total of 24 subjects from the control group and 21 subjects from the HBOT group were included in the cardia MRI analysis.

The baseline characteristics and comparability of the cohort are provided in Table 1. There were no significant differences between groups in anthropometric measurements, body composition or baseline VO_2_Max or VO_2_Max/Kg (Table 2).

**Table 1:** Participants’ characteristics

			All (*N* = 63)		Control (*N* = 33)		HBOT (*N* = 30)	
Age (years)			69.70 ± 3.59		68.81 ± 3.34		70.68 ± 3.64	
Males			39 (61.9%)		23 (69.7%)		16 (53.3%)	
Aerobic Hours/Week			2.00 ± 2.37		2.27 ± 2.68		1.71 ± 1.98	
Previous orthopedic surgery			23 (36.5%)		12 (36.3%)		11 (36.6%)	
Chronic medical conditions								
	Atrial fibrillation		4 (6.3%)		0 (0%)		4 (13.3%)	
	Hypothyroidism		7 (11.1%)		3 (9.1%)		4 (13.3%)	
	Obstructive sleep apnea		3 (4.8%)		0 (0%)		3 (10%)	
	Asthma		3 (4.8%)		2 (6.1%)		1 (3.3%)	
	Benign prostatic hyperplasia		14 (22.2%)		7 (23.3%)		7 (21.2%)	
	Gastroesophageal reflux		5 (7.9%)		2 (6.1%)		3 (10%)	
	Osteoporosis		10 (15.9%)		5 (15.2%)		5 (16.7%)	
	Rheumatic arthritis		4 (6.3%)		3 (9.1%)		1 (3.3%)	
	Osteoarthritis		11 (17.5%)		4 (12.1%)		7 (23.3%)	
	Diabetes mellitus		10 (15.9%)		7 (21.2%)		3 (10%)	
	Hypertension		14 (22%)		7 (21.2%)		7 (23.3%)	
	Dyslipidemia		30 (47.6%)		14 (42.4%)		16 (53.3%)	
	Ischemic heart disease		6 (9.5%)		4 (12.1%)		2 (6.7%)	
	History of smoking		24 (38.1%)		14 (42.4%)		10 (33.3%)	
	Smoking pack years		22.38 ± 13.33		21.21 ± 10.75		24.0 ± 16.79	
	Quit smoking years		23.96 ± 12.36		23.71 ± 11.86		24.3 ± 13.68	
Chronic medications								
	Anti-aggregation		14 (22.2%)		6 (18.2%)		8 (26.7%)	
	ACE-inhibitors/ ARB blockers		14 (22.2%)		8 (24.2%)		6 (20%)	
	Beta Blockers		11 (17.5%)		6 (18.2%)		5 (16.7%)	
	Alpha Blockers		13 (20.6%)		6 (18.2%)		7 (23.3%)	
	Calcium blockers		6 (9.5%)		3 (9.1%)		3 (10%)	
	Diuretics		3 (4.8%)		1 (3%)		2 (6.7%)	
	Statins		19 (30.2%)		9 (27.3%)		10 (33.3%)	
	Oral hypoglycemics		5 (7.9%)		4 (12.1%)		1 (3.3%)	
	Proton pump inhibitors		7 (11.1%)		4 (12.1%)		3 (10%)	
	Hormones		4 (6.3%)		1 (3%)		3 (10%)	
	Benzodiazepines		9 (14.3%)		6 (18.2%)		3 (10%)	
	SSRI		8 (12.7%)		3 (9.1%)		5 (16.7%)	

ACE = Angiotensin converting enzyme, ARB = Angiotensin II receptor blockers, SSRI = selective serotonin reuptake inhibitors

**Table 2:** Baseline measurements

			All (*N* = 63)		Control (*N* = 33)		HBOT (*N* = 30)		Sig
Height			169.16 ± 9.13		169.90 ± 9.12		168.33 ± 9.23		0.499
Weight			76.39 ± 11.91		77.36 ± 12.11		75.31 ± 11.79		0.500
BMI			26.63 ± 3.24		26.70 ± 3.08		26.54 ± 3.46		0.843
BSA			1.87 ± 0.18		1.88 ± 0.19		1.86 ± 0.16		0.712
Fat mass%			29.40 ± 7.52		28.51 ± 7.18		30.38 ± 0.7.89		0.328
VO_2_Max			1514.62 ± 521.17		1584.69 ± 493.87.		1437.53 ± 547.57		0.266
VO_2_Max/Kg			19.73 ± 5.93		20.64 ± 5.76		18.72 ± 6.05		0.202

### Physical performance evaluation

**Fig. 2:**
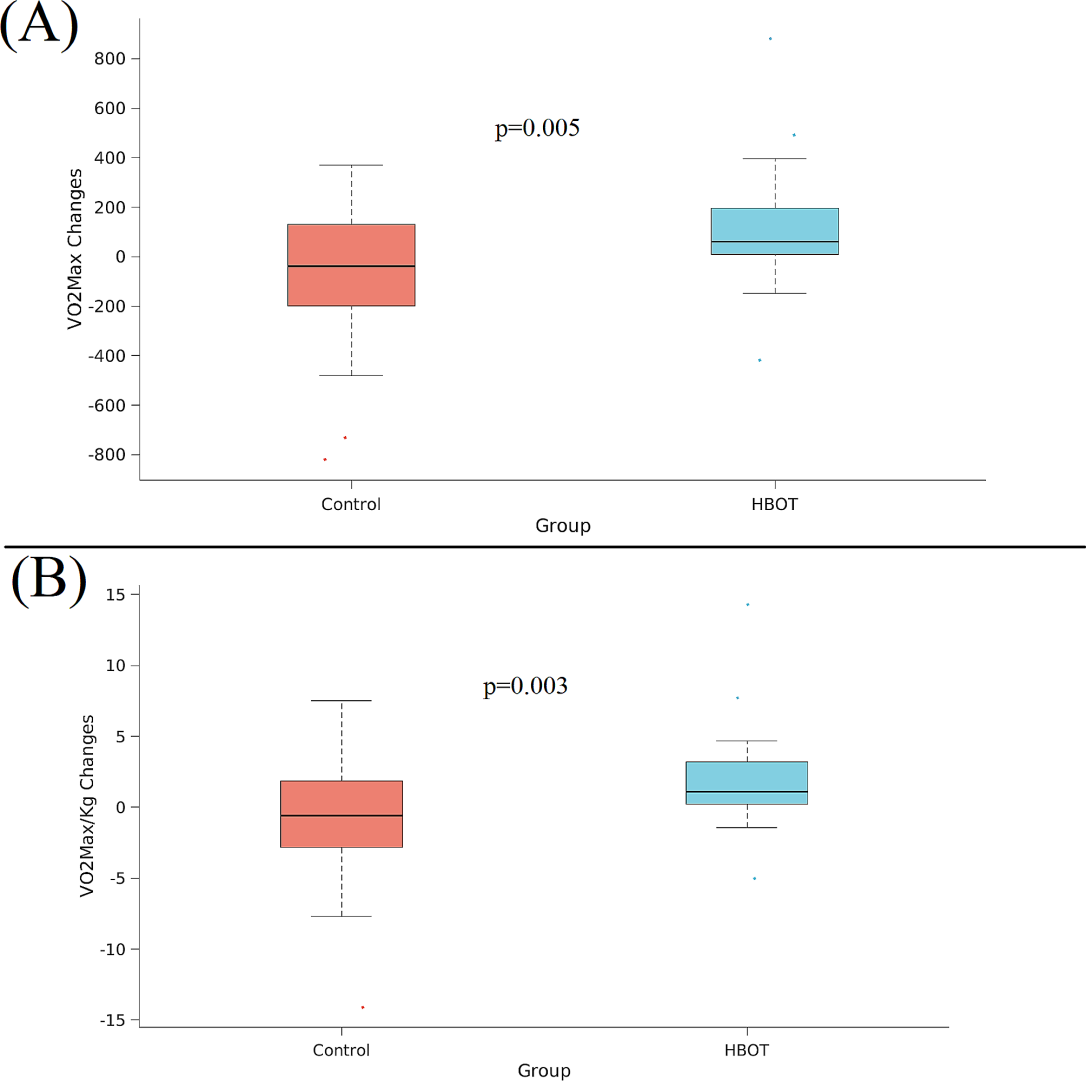
VO_2_Max and VO_2_Max/Kg significant changes The CPET parameters, (A) VO_2_Max and (B) VO_2_Max/Kg, shown in boxplots, where the central mark indicates the median and the bottom and top edges of the box indicate the 25th and 75th percentiles, respectively

**Fig. 3:**
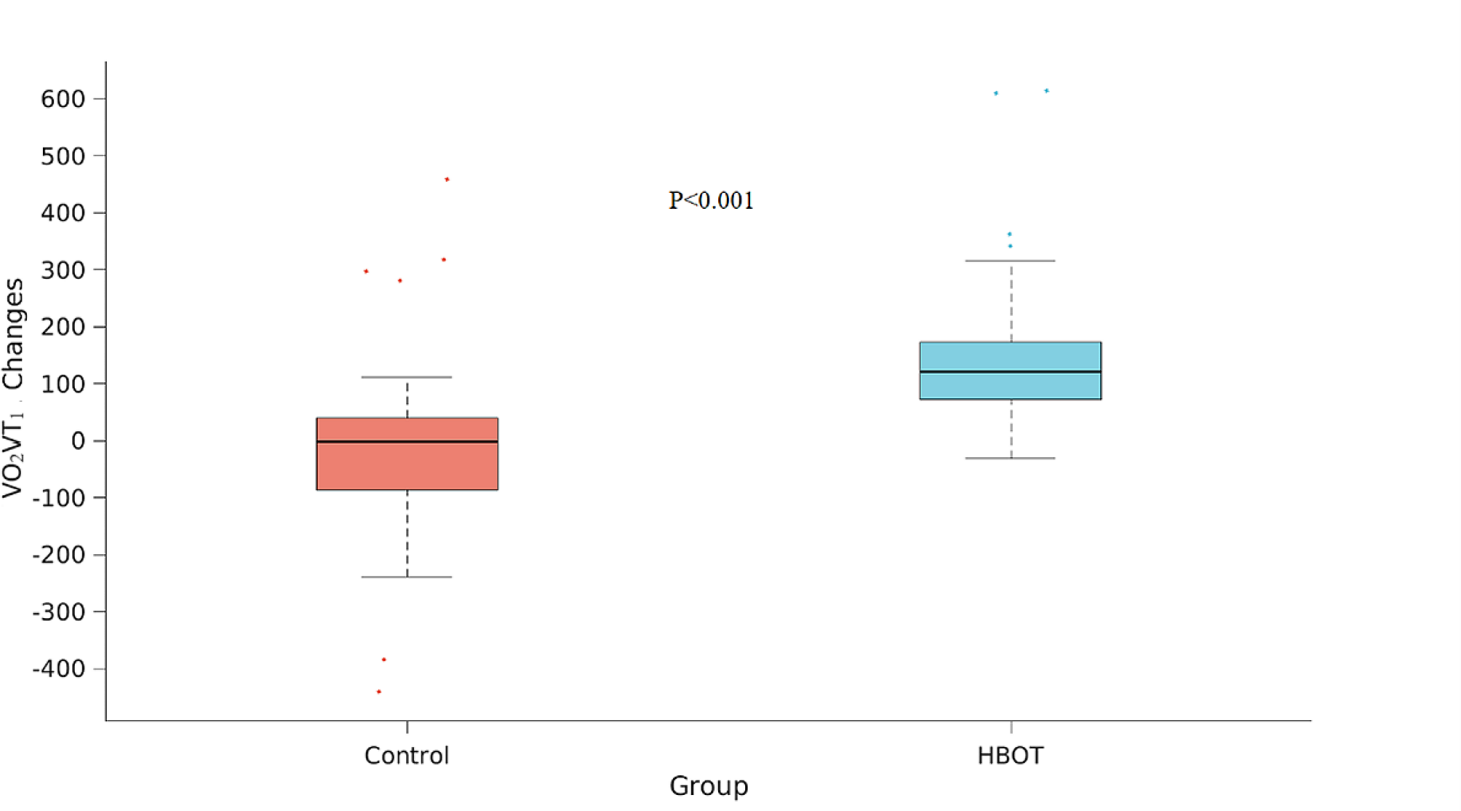
VO_2_VT_1_ significant changes The CPET parameters, shown in boxplots, where the central mark indicates the median and the bottom and top edges of the box indicate the 25th and 75th percentiles, respectively

There were no significant differences between the groups in CPET parameters at baseline. Following HBOT, there was a significant increase in VO_2_Max (1437.53 ± 57 ml/min to 1548.93 ± 538.62 ml/min, ANCOVA F = 8.508, *p* = 0.005) with a net Cohen’s d effect size of 0.3410 compared to the control (Table 2). Similar results were demonstrated in VO_2_Max/kg with a net Cohen’s d effect size of 0.455 (ANCOVA, F = 9.450, *p* = 0.003) (Table 3; Figure 2). Oxygen consumption measured at the first ventilatory threshold (VO_2_VT_1_) was significantly increased in the HBOT group (767.53 ± 217.71 ml/min to 927.57 ± 308.28 ml/min, ANCOVA, F = 11.561, *p* = 0.001) with a net Cohen’s d effect size of 0.617 compared to the control group (Table 3; Figure 3).

**Table 3:** CPET changes

	Control (*N* = 33)			HBOT (*N* = 30)					
	Baseline	Control		Baseline	HBOT		Baseline Comparison *P*-value	Net Effect Size	ANCOVA– Group F(*P*)
**Primary Endpoint**									
VO_2_Max (mL/min)	1584.70 ± 493.87	1517.98 ± 488.42		1437.53 ± 547.57	1548.93 ± 538.62		0.269	0.341	**8.508(0.005)**
VO_2_Max/Kg (mL/kg/min)	20.64 ± 5.76	19.84 ± 5.71		18.72 ± 6.05	20.63 ± 6.08		0.202	0.455	**9.450 (0.003)**
**Secondary Endpoints**									
Power (watts)	129.21 ± 51.77	130.96 ± 58.21		117.50 ± 46.95	122.17 ± 47.08		0.352	0.068	0.184 (0.670)
VO_2_VT_1_ (mL/min)	841.25 ± 227.12	837.60 ± 191.17		767.53 ± 217.71	927.57 ± 308.28		0.194	0.617	**11.561 (0.001)**
VO_2_/WR Slope	8.39 ± 2.09	8.62 ± 2.12		8.45 ± 1.61	8.59 ± 1.37		0.904	0.121	0.001 (0.978)
VO_2_/HR	11.99 ± 3.89	11.55 ± 3.48		10.96 ± 3.78	11.55 ± 3.48		0.292	0.179	1.155 (0.287)
VE/VCO_2_ Slope	30.83 ± 5.08	28.89 ± 6.15		28.66 ± 3.83	28.34 ± 4.34		0.064	0.263	0.000(0.994)
BR (%)	49.47 ± 17.55	50.24 ± 23.74		45.09 ± 19.74	43.42 ± 16.96		0.442	-0.105	1.748 (0.193)
RQ	1.14 ± 0.09	1.13 ± 0.09		1.11 ± 0.11	1.14 ± 0.02		0.236	0.369	1,668 (0.202)

VO_2_Max = maximal oxygen consumption, VO_2_VT_1_ = oxygen consumption at the first ventilatory threshold, Kg = Kilograms, BR = breathing reserve

*P* < 0.05 in bold

There were no significant differences in power, BR, VO_2_/HR, VO_2_/WR slope, VE/VCO_2_ and RQ (Table 3).

### Cardiac perfusion

At baseline, there were no differences in either global MBF and MBV between the groups (Table 4). Following HBOT, there was a significant increase in global MBF (0.34 ± 0.10 ml/100 g/min to 0.42 ± 0.19 ml/100 g/min), with a large net effective size of 0.797, compared to the control group (ANCOVA F = 7.686, *p* = 0.008). Similarly, the HBOT group had an increased global MBV (0.53 ± 0.14 ml/100 g to 0.61 ± 0.22 ml/100 g) with a large net effect size of 0.896, compared to the controlled group (ANCOVA F = 7.458, *p* = 0.009) (Table 4).

**Table 4:** Cardiac perfusion changes

	Control (*N* = 25)			HBOT (*N* = 21)					
	Baselin4e	Control		Baseline	Post-HBOT		Baseline Comparison *P*-value	Net Effect Size	ANCOVA– Group F(*P*)
MBF	0.32 ± 0.08	0.30 ± 0.08		0.34 ± 0.10	0.42 ± 0.19		0.458	0.797	**7.686 (0.008)**
MBV	0.51 ± 0.08	0.46 ± 0.12		0.53 ± 0.14	0.61 ± 0.22		0.580	0.896	**7.458 (0.009)**

MBF = myocardial blood flow, normalized, MBV = myocardial blood volume, normalized

*P* < 0.05 in bold

There were moderate correlations between global MBV changes and VO_2_Max (*r* = 0.45, *p* = 0.043) and VO_2_Max/kg changes (*r* = 0.45, *p* = 0.041). There were strong correlations between global MBV and VO_2_/WR slope (*r* = 0.73, *p* < 0.001) and VO_2_/HR (*r* = 0.72, *p* < 0.001).

### Pulmonary function

No significant differences were noted in pulmonary function measurements between the groups at baseline. There were no significant changes following HBOT or control interventions (Table 5).

**Table 5:** Pulmonary function changes

	Control (*N* = 33)			HBOT (*N* = 30)					
	Baseline	Control		Baseline	Post-HBOT		Baseline Comparison *P*-value	Net Effect Size	ANCOVA– Group F(*P*)
FEV1 (liters)	2.72 ± 0.58	2.66 ± 0.58		2.51 ± 0.66	2.49 ± 0.65		0.193	-0.068	0.214 (0.646)
FVC1 (liters)	3.22 ± 0.68	3.17 ± 0.73		3.01 ± 0.74	2.95 ± 0.64		0.235	-0.004	0.143 (0.707)
FEV1/FVC	0.84 ± 0.06	0.85 ± 0.06		0.84 ± 0.08	0.84 ± 0.07		0.672	0.027	0.003 (0.957)
FEF25-75 (liters)	3.12 ± 0.92	3.08 ± 0.93		2,91 ± 1.07	2.94 ± 1.12		0.396	0.079	0.053 (0.818)

FEV1 = forced expiratory volume in one second, FVC = forced vital capacity, FEF25-75 = percentage of the predicted value for forced expiratory flow at 25–75% of forced vital capacity, PEF = pulmonary expiratory flow

*P* < 0.05 in bold

### Safety

Three participants (11.1%) experienced mild middle ear barotrauma (TEED 1–2) in the HBOT group compared to none (0%) in the control group. All events were treated conservatively, and all participants completed their protocol. Fifteen participants (55%) had visual acuity changes in the HBOT group, compared to ten (34.4%) in the control group. Three participants (11.1%) in the HBOT group, compared to seven (24.1%) in the control group had cataract level acceleration.

## Discussion

We have found that HBOT can significantly enhance physical performance in the elderly. Both VO_2_Max, VO_2_Max/Kg andVO_2_VT_1_ significantly increased following HBOT compared to the control group. Moreover, the HBOT group had a significantly increased general cardiac perfusion, measured by both MBF and MBV.

While the impact of VO_2_Max on daily life is generally minimal for young individuals, elderly individuals heavily rely on their VO_2_Max to perform everyday tasks effectively [25]. Thus, the age-related non-linear decline in VO_2_Max [7], has a clear significance on the capacity of maintaining an independent lifestyle. The first ventilatory threshold (VO_2_VT_1_) is considered a superior indicator of performance compared to VO_2_Max, as a higher VT_1_ (first ventilatory threshold) suggests a greater ability to sustain intensity without experiencing acidosis or the accumulation of lactate [26]. Moreover, VT_1_ can be objectively measured during a cardiopulmonary exercise test (CPET), independent of the participant’s motivation, which can potentially influence VO_2_ peak [27]. In our study, we utilized the first ventilatory threshold as a reliable estimation of blood lactate levels, eliminating the need for multiple needle pricks and lengthier testing protocols during CPET [20]. Notably, our findings demonstrate that HBOT prominently enhances oxygen consumption at the first ventilatory threshold as well as the maximal VO_2_ consumption.

VO_2_Max is determined by both the capacity for oxygen delivery and for oxygen utilization, mostly by muscles [7]. Considering delivery, previous studies have shown older adults have lower oxygen delivery that limits the rate at which the muscles increase oxygen consumption [7]. The restriction in blood flow may be attributed to reduced maximal cardiac output or altered cardiac output distribution.

Blood vessels become thicker and stiffer with aging, which in turn increases blood flow resistance and increases cardiac afterload [28]. More so, age-related endothelium injury results in decreased nitric oxide production, which further reduces blood flow [28]. Left ventricular contractility and ejection fraction also decrease with age due to cardiomyocytes age-related necrosis as well as increased afterload secondary hypertrophy [29]. In our previous study, performed on the same elderly population, demonstrated that prolonged HBOT protocol increases left ventricular and right ventricular systolic function, and improves myocardial performance [19]. In the current study, for the first time, we demonstrate HBOT can increase cardiac perfusion as well which may be both a contributing factor or the result of improved cardiac function.

Assessing the extent of myocardial perfusion is a critical measure of the heart’s ability to deliver oxygen. Historically, positron emission tomography (PET) and single photon emission computed tomography (SPECT) have been the preferred methods for estimating myocardial perfusion. However, cardiac MRI, which is a non-invasive imaging technique free of radiation, is now being increasingly utilized for evaluating and quantifying myocardial perfusion [30–32]. MBF quantification reflects coronary microcirculation in the absence of obstructive coronary artery disease. MBV is the volume of blood residing in myocardial vessels, 90% of which is in capillaries. In the current study, HBOT increases both MBF and MBV, which may suggest improved cardiac microcirculation. The beneficial effect of HBOT on microvasculature has been thoroughly studied in non-healing peripheral wounds, radiation injury as well as critical organs such as the brain [17, 33–35, 18]. Specifically, in our previous study on the same aging population, cerebral blood flow and cerebral blood volume were increased and angiogenesis was demonstrated by skin biopsies [17, 18].

The HBOT protocol utilized in the current study involved repeated intermittent exposure to high oxygen levels, known as the hyperoxic hypoxic paradox [13]. These intermittent hyperoxic exposures induce physiological responses similar to those observed during hypoxia. HBOT triggers the release and enhances the stability and activity of hypoxia-inducible factors (HIFs), a group of transcription factors. Consequently, HIF-1α and HIF-2α regulate the release of vascular endothelial growth factor (VEGF), an angiogenic factor. VEGF is widely recognized as the key regulator of angiogenesis, promoting the migration of endothelial progenitor cells from the bone marrow to the circulatory system, recruiting endothelial cells from existing blood vessels, and facilitating the formation of new blood vessels [36, 37]. Notably, these circulating angiogenic cells migrate to areas of ischemia, where they contribute to vascular remodeling and stimulate angiogenesis and improve microcirculation [38]. Based on these observations, we propose that repetitive fluctuations in oxygen levels may potentially enhance regional cerebral blood flow and cognitive functions in elderly individuals.

Considering the second determinant of VO_2_Max, oxygen utilization, age-related reduction in mitochondrial oxidative capacity and/or lesser extent in mitochondria volume have been vastly reported [7, 39–42]. In our previous study on middle age athletes using muscle biopsies, 40 intermittent exposures to HBOT induced both mitochondrial oxidative capacity as well as biogenesis, the increase in mitochondrial content [16]. Although, this hasn’t been directly evaluated, mitochondrial function and volume increase may explain some of the performance enhancement induced in the elderly population in the current study.

Physical training, in particular, high intensity interval training, has been shown to improve cardiovascular function in older adults with significant increase in VO_2_Max [43, 44]. More so, a reduction or discontinuation of training results in a more rapid decline in VO_2_Max compared to the gradual decline associated with aging alone in elderly master athletes. This can quickly negatively affect the benefits gained from previous long-term training. On the other hand, resuming exercise training has the potential to rapidly restore some of the lost VO_2_Max and exercise performance [45]. This study demonstrates, for the first time in humans, a significant increase in VO_2_Max solely due to HBOT alone in the elderly. One can anticipate that combining physical training and HBOT will have synergistic effects but that remains to be investigated.

The present study has several limitations that should be acknowledged. Firstly, the sample size was relatively small, which may have reduced the sensitivity of the study. However, the fact that significant changes were observed through rigorous statistical analyses within this small group suggests a relatively strong impact of the intervention. Secondly, the control group was a non-intervention group rather than a sham-intervention group. While the outcome assessors were objective and blinded, the participants themselves were aware of their group assignment. Thirdly, the long-term duration of the observed effects remains to be determined and requires further investigation through follow-up studies. Fourthly, direct evaluation of mitochondrial function through muscle biopsies was not conducted. Fifthly, the optimal number of sessions and the specific protocol for each session have yet to be established. Despite these limitations, several strengths of the study should be emphasized. The isolated effect of HBOT was measured by monitoring both groups for any potential lifestyle changes (such as diet and exercise), medication usage, or other interventions that could have acted as confounding factors. Furthermore, various assessments including pulmonary function tests, body composition analysis, and blood tests were conducted to control for possible confounding factors. Lastly, both CPET and cardiac MRI were conducted more than 1 week after the last HBOT session further supports the conclusion that repeated HBOT induces significant biological changes rather than transient due to increased oxygen delivery.

## Conclusions

The study findings suggest that the newly used HBOT can enhance physical performance in aging adults. The key enhancements observed include improvements in maximal oxygen consumption, and the first ventilatory threshold. Moreover, the use of cardiac MRI demonstrated increased cardiac perfusion as a significant mechanism underlying the observed improvements induced by HBOT.

## Data Availability

The datasets used and/or analyzed during the current study are available from the corresponding author on reasonable request.
